# Mixed Neuroendocrine Non-Neuroendocrine Neoplasm of the Ampulla of Vater: Report of a Rare Location

**DOI:** 10.31486/toj.25.0030

**Published:** 2025

**Authors:** Ankit Rai, Vaibhav Kumar Varshney, Peeyush Varshney, Lokesh Agarwal, Meenakshi Rao, Ayushi Agarwal

**Affiliations:** ^1^ Department of Surgical Gastroenterology, All India Institute of Medical Sciences, Jodhpur, India; ^2^ Department of Pathology, All India Institute of Medical Sciences, Jodhpur, India; ^3^ Department of Radiology, All India Institute of Medical Sciences, Jodhpur, India

**Keywords:** *Adenocarcinoma*, *ampulla of Vater*, *neuroendocrine tumors*, *pancreaticoduodenectomy*, *robotic surgical procedures*

## Abstract

**Background:**

Mixed neuroendocrine non-neuroendocrine neoplasms (MiNENs) are rare tumors of the gastrointestinal tract with neuroendocrine and non-neuroendocrine components. Ampullary MiNENs are extremely rare, with few cases reported to date.

**Case Report:**

A 41-year-old male was diagnosed incidentally with a dilated common bile duct and intrahepatic biliary radicles while being evaluated for right ureteric calculi. Contrast-enhanced computed tomography scan of the abdomen showed a mass in the ampullary region with a positive double duct sign. Side-viewing endoscopy indicated an ampullary growth, and biopsy confirmed adenocarcinoma. The patient underwent total robotic pancreatoduodenectomy with an uneventful postoperative course. His final histopathologic examination revealed a tumor with 2 components, each of which accounted for at least 30% of the tumor: a neuroendocrine tumor and an adenocarcinoma with signet ring cells. The patient received adjuvant chemotherapy and at 1-year follow-up showed no evidence of recurrence.

**Conclusion:**

Ampullary MiNENs are rare composite gastroenteropancreatic tumors characterized by histologic heterogeneity; they can be effectively treated with robotic pancreatoduodenectomy. The more aggressive component of the MiNEN should be considered when determining an adjuvant therapy.

## INTRODUCTION

Mixed neuroendocrine non-neuroendocrine neoplasms (MiNENs) are rare epithelial gastroenteropancreatic tumors with both neuroendocrine and non-neuroendocrine histologies in varying proportions. Based on the histologic features and the distribution of the individual components, MiNENs are classified as composite tumors (intimately intermingled within the tumor mass), collision tumors (separate, juxtaposed areas of tumor mass), or amphicrine tumors (coexisting at the cellular level).^1^

According to the 2017 World Health Organization (WHO) definition, MiNENs should contain at least 30% each of neuroendocrine and non-neuroendocrine components, because the biological behavior of the individual component may be insignificant if it accounts for <30% of the tumor.^2^ More than 50% of MiNENs occur in the colorectum, with nearly 60% reported in the appendix.^3^ The incidence is much lower elsewhere in the gastrointestinal tract: 5.9% to 15.9% in the esophagus, 6% to 20% in the stomach, and <1% in the small intestine.^3-5^ In the pancreatobiliary region, MiNENs account for 0.5% to 2% of all pancreatic tumors and 2% of hepatobiliary tumors.^4^ Ampullary MiNENs are extremely rare, with, to our knowledge, 21 previously reported cases.^6-9^

We report a case of MiNEN located at the ampulla of Vater that was treated with robotic pancreatoduodenectomy.

## CASE REPORT

A 41-year-old male presented for evaluation of symptomatic right ureteric calculi. He was managed with ureteroscopic lithotripsy and percutaneous nephrolithotomy. Ultrasound assessment showed that the patient's bile duct and bilobar intrahepatic ducts were dilated, and he had an ampullary mass. The patient had no symptoms of jaundice, fever, gastrointestinal bleeding, anorexia, or weight loss. His liver biochemistry was within normal range, and serum tumor markers were also normal: CA 19-9 was 1.10 U/mL (reference value, <37 U/mL) and carcinoembryonic antigen was 2.277 ng/mL (reference range, 0-4 ng/mL).

**Figure 1.**
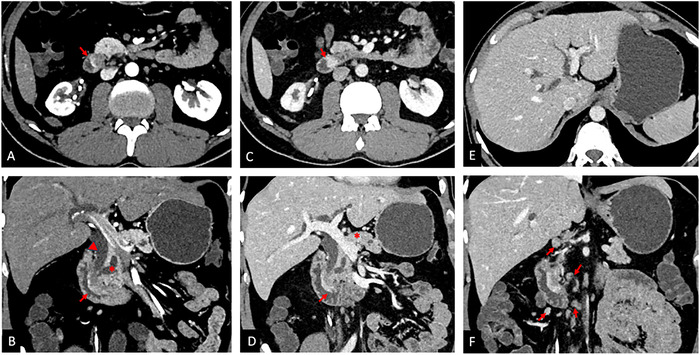
Contrast-enhanced computed tomography (A) axial reformatted image in pancreatic phase shows an ill-defined mass in the ampullary region with peripheral rim enhancement (arrow). (B) Coronal reformatted image in pancreatic phase shows a smooth bulge into the medial wall of the second part of the duodenum (arrow), as well as cutoff of the distal common bile duct (arrowhead) and pancreatic duct (asterisk) with upstream dilatation (the double duct sign). (C) Axial and (D) coronal reformatted images in portal venous phase show progressive enhancement of the mass (arrows), with the mass appearing iso-hyperenhancing to the pancreatic parenchyma (asterisk in image D). (E) Axial image in portal venous phase shows bilobar intrahepatic biliary dilatation. No focal lesions were noted in the liver parenchyma. (F) Coronal reformatted image in portal venous phase shows heterogeneously enhancing subcentimetric, periportal, peripancreatic, and mesenteric lymph nodes (arrows).

Contrast-enhanced computed tomography (CT) of the abdomen and pelvis conducted according to the pancreatic protocol showed a 28 × 11-mm ill-defined mass in the region of the ampulla that was bulging into the medial wall of the second part of the duodenum. The mass was obstructing the ampulla and causing abrupt cutoff of the distal common bile duct and main pancreatic duct with upstream extrahepatic and bilobar intrahepatic biliary dilatation. The dilatation of the common bile duct and pancreatic duct secondary to distal block manifests as the double duct sign. Peripheral rim enhancement of the mass was noted in pancreatic phase, and progressive homogeneous enhancement was observed in portal venous phase. Heterogeneously enhancing subcentimetric, periportal, peripancreatic, and mesenteric lymph nodes were noted with no imaging evidence of distant disease. Imaging features favored periampullary adenocarcinoma; however, in view of the peripheral rim of arterial enhancement and smooth bulge along the duodenum, a differential diagnosis of neuroendocrine tumor was considered (Figure 1). Ampullary biopsy performed by side-viewing endoscopy revealed adenocarcinoma.

**Figure 2.**
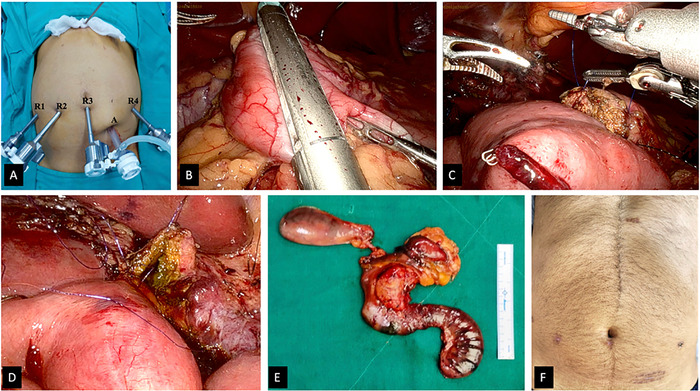
Intraoperative images show (A) port placement for the robotic pancreatoduodenectomy, (B) stomach transection using the robotic SureForm stapler (Intuitive Surgical, Inc), (C) duct-to-mucosa pancreaticojejunal anastomosis, (D) hepaticojejunal anastomosis, (E) pancreatoduodenectomy surgical specimen, and (F) postoperative abdomen with minimal scars.

The patient underwent robotic pancreatoduodenectomy. After systematic vascular ligation, pancreaticojejunostomy was performed by the duct-to-mucosa technique (Figure 2). The patient's postoperative stay was uneventful, and he was discharged on postoperative day 5.

**Figure 3.**
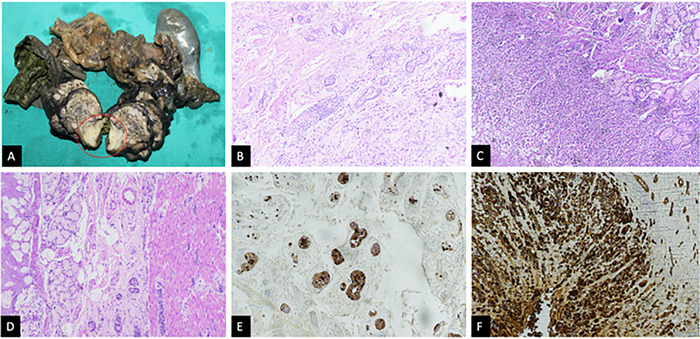
(A) Cut section of the gross pancreatoduodenectomy specimen shows the ampullary growth circled in red. (B) Low-power view of the periampullary tumor shows the neuroendocrine tumor component (composed of well-formed nests of monomorphic cells) in the upper left half of the image and the adenocarcinoma (with signet ring cell components) in the lower right corner of the image (hematoxylin and eosin [H&E] stain, magnification ×100). (C) Low-power view shows the adenocarcinoma with signet ring cells in the duodenal mucosa, with entrapped benign duodenal glands in between (H&E stain, magnification ×100). (D) The duodenal wall also shows a well-differentiated neuroendocrine tumor composed of nests of monomorphic cells in the muscle layer on the right side of the image (H&E stain, magnification ×100). (E) Chromogranin A immunohistochemistry demonstrates chromogranin A positivity in the neuroendocrine tumor component. (F) Immunohistochemistry for the pancytokeratin cocktail (AE1/AE3) highlights the infiltrating signet ring cells in the duodenal mucosa.

Gross pathologic examination showed a 28 × 16 × 14-mm unifocal mass arising from the ampullary region. Microscopic examination revealed an invasive tumor originating from the ampulla of Vater that was composed of 2 components. One component, which accounted for 60% of the total tumor, consisted of organoid nests, glands, and trabeculae of monomorphic neuroendocrine cells with round nuclei and stippled chromatin. On immunohistochemistry, tumor cells were positive for chromogranin A and synaptophysin, with a Ki-67 labeling index of 15%, overall indicating characteristics of a neuroendocrine tumor, grade 2. The duodenal wall also showed a well-differentiated neuroendocrine tumor composed of nests of monomorphic cells in the muscle layer. The second component, constituting 40% of the total tumor showed adenocarcinoma with signet ring cells in the duodenal mucosa, with entrapped benign duodenal glands in between. The duodenal lining over the tumor was partially ulcerated. The tumor was directly invading the muscularis propria of the duodenum, with involvement of 3 mm of pancreatic parenchyma, displaying features of a poorly differentiated adenocarcinoma with signet ring cells. Immunohistochemistry for pancytokeratin cocktail (AE1/AE3) confirmed the infiltrating signet ring cells in the duodenal mucosa (Figure 3). All resection margins were negative for dysplasia, intraepithelial neoplasia, and invasive carcinoma. Two lymph nodes tested positive for tumor deposits; both contained deposits of neuroendocrine tumor. According to the 8th edition of the *TNM Classification of Malignant Tumours*, the stage was pT3aN1.^10^

The patient subsequently received 4 cycles of adjuvant chemotherapy with gemcitabine injection 1,400 mg/m^2^ on days 1, 8, and 15 and cisplatin injection 50 mg/m^2^ on days 1 and 15. At 1-year follow-up, the patient was doing well without any evidence of recurrence.

## DISCUSSION

WHO introduced the term MiNEN in 2017 to describe tumors with both neuroendocrine and non-neuroendocrine components^2^ and was first used to describe tumors of pancreatic origin; however, in the 2019 update, the term was expanded to include all tumors of the gastrointestinal tract with neuroendocrine and non-neuroendocrine components and meeting the defining criteria.^11^ MiNENs occur more commonly in males and have an incidence of 1.225/1,000,000 person-years.^4^

MiNENs of ampullary origin usually present with nonspecific symptoms, such as vague abdominal discomfort, pain, nausea, or vomiting. Although MiNENs have a neuroendocrine component, endocrine symptoms are generally mild or absent.^12^ In our case, the patient was asymptomatic, perhaps because the ampullary lesion was small and was detected early.

To identify and stage the lesion, imaging evaluation is performed with the pancreatic protocol contrast-enhanced CT. Because MiNENs contain neuroendocrine and non-neuroendocrine components, the imaging features mimic a neuroendocrine tumor and an adenocarcinoma and must be differentiated. Morphologically, MiNENs tend to present as larger tumors compared to neuroendocrine tumors and pancreatic ductal adenocarcinomas because in their early stages, MiNENs often do not present with the typical symptoms seen in pancreatic ductal adenocarcinoma, and as a result, can grow substantially before causing compressive symptoms that lead to clinical detection. Neuroendocrine tumors have well-defined margins compared with the ill-defined margins of adenocarcinomas and MiNENs.^13^ On contrast administration, neuroendocrine tumors show marked arterial enhancement with iso-hyperenhancement in delayed phase images in contrast to pancreatic adenocarcinomas that are hypoenhancing in arterial phase images and show progressive enhancement because of contrast retention on delayed phase images. MiNENs are heterogeneous tumors because of the mixed solid cystic components and show variable arterial enhancement, with progressive enhancement in portal venous phase. Pancreatic ductal adenocarcinomas have a mild degree of enhancement, MiNENs have a moderate degree of enhancement, and neuroendocrine tumors have a marked degree of enhancement.^13^ Among these features, moderate enhancement has the highest specificity for predicting MiNENs.^14^ MiNENs exhibit local infiltration; however, vascular involvement is less common in MiNENs compared to pancreatic ductal adenocarcinomas, likely because MiNENs are not as invasive as pancreatic ductal adenocarcinomas.^13^

Histopathologically, a MiNEN diagnosis requires that each component—the neuroendocrine and non-neuroendocrine—accounts for at least 30% of the tumor. This arbitrary cutoff is established on the assumption that prognosis depends on the dominant component, especially in metastatic disease.^15^ Nonetheless, this cutoff has drawbacks and lacks validation in extensive systematic reviews.^16^ Moreover, the one-third composition requirement for diagnostic criteria may overlook the detrimental effects of a smaller component that fails to meet this threshold and consequently may lead to underreporting of the actual incidence, as strict quantification standards might not be satisfied in preoperative random biopsy samples. Furthermore, it is crucial that the diagnosis be made on patients who have not undergone neoadjuvant therapy, as research has indicated that neuroendocrine cells may proliferate following systemic therapy in various adenocarcinomas.^17^

The histopathologic findings of these lesions require immunohistochemistry confirmation. The most commonly used markers for the neuroendocrine component are CD56, chromogranin A, and synaptophysin. Other markers include somatostatin receptor subtype 2A, insulinoma-associated protein 1, and CD57. Immunohistochemistry markers for non-neuroendocrine components depend on the histologic subtype, with adenocarcinomas expressing cytokeratin 19, cytokeratin 7, carcinoembryonic antigen, and CA 19-9.^3^ However, diagnosis of a MiNEN by immunohistochemistry-based quantification alone is insufficient because neuroendocrine markers may be present in non-neuroendocrine tumors, and discrete histopathologic findings of each morphology must be present.

MiNENs are aggressive tumors, with the majority harboring high-grade neuroendocrine tumors and thus associated with poor long-term survival.^18^ Because of the rarity of a MiNEN diagnosis, consensus regarding the best treatment is lacking. The biological behavior of these lesions is not necessarily an average of the individual components but rather a sum of the components, as each may progress independently. Given the aggressive nature of MiNENs, multimodal treatment incorporating systemic chemotherapy and radical resection is warranted.^19^ For patients with resectable MiNENs, curative-intent surgery is the first line of management. Even for patients with high-grade MiNENs, surgery is the first-line treatment as these tumors are less aggressive than pure neuroendocrine carcinomas. Pancreatoduodenectomy is the standard surgery for ampullary MiNENs. Surjan et al first reported robotic pancreaticoduodenectomy for an ampullary MiNEN in 2024.^20^ To our knowledge, we report the second case in the literature of robotic pancreaticoduodenectomy for an ampullary MiNEN.

Although large-scale studies evaluating the role of neoadjuvant and adjuvant therapy in patients with MiNEN are lacking, adjuvant therapy is generally considered postresection, but whether the therapy should target the neuroendocrine or non-neuroendocrine component remains unclear. Some authors have suggested that the therapy should target the dominant histologic component, as it generally determines the overall outcome.^21-23^ Our patient had a MiNEN with a poorly differentiated exocrine component along with multifocal lymphovascular and perineural invasion. We chose chemotherapy to target the more aggressive tumor component. More than 80% of patients with ampullary MiNEN present with localized disease, with a median survival of 39 months for localized and 11 months for advanced disease.^24^

## CONCLUSION

MiNENs are rare composite gastroenteropancreatic tumors with neuroendocrine and non-neuroendocrine components and histologic heterogeneity. The more aggressive component of the MiNEN should be considered when deciding on an appropriate management strategy.

## Notes

*This article meets the Accreditation Council for Graduate Medical Education and the American Board of Medical Specialties Maintenance of Certification competencies for Patient Care and Medical Knowledge.*
